# Potential Antidiabetic Activity of *Apis mellifera* Propolis Extraction Obtained with Ultrasound

**DOI:** 10.3390/foods13020348

**Published:** 2024-01-22

**Authors:** Javier A. Hernández-Martínez, Armando Zepeda-Bastida, Irma Morales-Rodríguez, Fabián Fernández-Luqueño, Rafael Campos-Montiel, Stephanie E. Hereira-Pacheco, Gabriela Medina-Pérez

**Affiliations:** 1 ICAP—Institute of Agricultural Sciences, Autonomous University of the State of Hidalgo, Tulancingo de Bravo 43000, Hidalgo, Mexico; j.a.h.m.jav05@hotmail.com (J.A.H.-M.); azepeda@uaeh.edu.mx (A.Z.-B.); irma_morales@uaeh.edu.mx (I.M.-R.); rcampos@uaeh.edu.mx (R.C.-M.); 2 Sustainability of Natural Resources and Energy Program, Cinvestav-Saltillo, Ramos Arizpe 25900, Coahuila, Mexico; fabian.fernandez@cinvestav.edu.mx; 3 Laboratorio de Interacciones Bióticas, Centro de Investigación en Ciencias Biológicas, Universidad Autónoma de Tlaxcala, Km 10.5 de la carretera San Martín Texmelucan, San Felipe Ixtacuixtla, Villa Mariano Matamoros 90120, Tlaxcala, Mexico; sehereirap@uatx.mx

**Keywords:** flavonoids, phenols, ɑ-glucosidase, ɑ-amylase, inhibition

## Abstract

Recent studies have linked phenolic compounds to the inhibition of digestive enzymes. Propolis extract is consumed or applied as a traditional treatment for some diseases. More than 500 chemical compounds have been identified in propolis composition worldwide. This research aimed to determine Mexican propolis extracts’ total phenolic content, total flavonoid content, antioxidant activity, and digestive enzyme inhibitory activity (ɑ-amylase and ɑ-glucosidase). In vitro assays measured the possible effect on bioactive compounds after digestion. Four samples of propolis from different regions of the state of Oaxaca (Mexico) were tested (Eloxochitlán (PE), Teotitlán (PT), San Pedro (PSP), and San Jerónimo (PSJ)). Ethanol extractions were performed using ultrasound. The extract with the highest phenolic content was PE with 15,362.4 ± 225 mg GAE/100 g. Regarding the flavonoid content, the highest amount was found in PT with 8084.6 ± 19 mg QE/100 g. ABTS^•+^ and DPPH^•^ radicals were evaluated. The extract with the best inhibition concentration was PE with 33,307.1 ± 567 mg ET/100 g. After simulated digestion, phenolics, flavonoids, and antioxidant activity decreased by 96%. In contrast, antidiabetic activity, quantified as inhibition of ɑ-amylase and ɑ-glucosidase, showed a mean decrease in enzyme activity of approximately 50% after the intestinal phase. Therefore, it is concluded that propolis extracts could be a natural alternative for treating diabetes, and it would be necessary to develop a protective mechanism to incorporate them into foods.

## 1. Introduction

Propolis, often called bee glue, is a substance that bees create to protect their hive. It serves various purposes, such as filling hive cavities, restricting entrance during cold days, and mummifying intruders to prevent decay. Bees collect plant resins and mix them with salivary enzymes and beeswax, forming propolis. Its composition varies across continents, regions, and plant species [1]. Propolis has been reported to have different biological properties, such as antioxidant, antidiabetic, antibacterial, antiviral, antifungal, and healing activities [2,3]. The compounds accountable for propolis’ functional characteristics can be grouped into phenolic acids, flavonoids, terpenes, aldehydes, fatty acids, stilbenes, and β-steroids. Compounds such as quercetin, chrysin, caffeic acid, cinnamic acid, and bacarin have antioxidant properties due to their ability to reduce oxidative stress by eliminating or neutralizing free radicals and chelating metal ions [4,5]. Oxidative stress has been related to different pathologies, including diabetes mellitus. Scientific reports show some medicinal plants utilized as antidiabetic elements in human nutrition [6]. Phytochemicals have been studied to prove their antidiabetic activity through different mechanisms, such as (1) stimulating insulin secretion, (2) performing insulin-like functions, and (3) decreasing glucose transport by inhibiting digestive enzymes. The enzymes ɑ-amylase and ɑ-glucosidase [7,8,9] are responsible for starch degradation, and when inhibited, it is possible to reduce the release of glucose molecules during digestion [10,11]. To predict changes in enzymatic mechanisms in the gastrointestinal tract, in vitro digestion systems are used to simulate the physical and biochemical conditions of the stomach and intestine [12,13]. This technique has proven to be a rapid and low-cost alternative to evaluate the interactions and bioavailability of bioactive compounds in the human body [14]. It has been reported that the diversity and quantity of chemical compounds in propolis depend on the geographical origin, type of solvent, and extraction method [15,16].

Regarding the geographical origin, the diversity and availability of flora, the climate, the year’s season, and the bee species are considered in addition to the technique and way of collecting raw propolis by beekeepers [17]. Water, methanol, chloroform, dichloromethane, and ethanol are the solvents used to extract propolis. Ethanol extracts are used more frequently for extractions due to their low price, non-toxic nature, and accessibility. Although water meets the above characteristics, its polarity prevents propolis from dissolving [18,19].

There are different extraction methods. Traditionally, extracts are obtained through maceration, which consists of mixing the propolis with the solvent and then letting it rest for a period that can range from 24 h to several weeks to increase the interaction between the solvent and the sample; however, extraction is more efficient when constant agitation is applied. New technologies, such as microwave-assisted extraction, ultrasound, and supercritical fluids, have been studied to improve the extraction mode [18,20,21,22]. Ultrasound consists of high-intensity, low-frequency sonic waves. In this way, it affects the physical, biochemical, and mechanical properties, which causes acoustic cavitation with the generation, growth, and collapse of bubbles. The frequency of ultrasound ranges between 20 and 100 kHz, and the intensity varies between 10 and 1000 W/cm^2^ [23,24,25,26]. This method is environmentally friendly, is low-cost, is reported to have better extraction yields, reduces process time, is highly selective, does not use high temperatures, and can be applied in combination with other methods, such as extraction using supercritical fluids [27,28,29]. The ultrasonic water bath is used through the transducer. It generates acoustic cavitation in the liquid medium randomly and constantly, releasing a large amount of energy through turbulence and mass transfer. It collapses the bubbles violently and with pressure against the propolis to favor the interaction with the solvent [30,31]. This study aimed to evaluate the inhibitory potential of ɑ-amylase and ɑ-glucosidase enzymes of bioactive compounds extracted with ultrasound from propolis under simulated digestion conditions.

## 2. Materials and Methods

### 2.1. Sample Collection

The propolis was collected from four different areas of the state of Oaxaca, Mexico: Eloxochitlán (PE), 18°10′34″ N 96°52′33″ W altitude 1345 m above sea level; Teotitlán (PT), 18°07′57″ N 97°04′20″ W altitude 1015 m above sea level; San Jerónimo (PSJ), 18°10’00″ N 96°55’00″ W altitude 1400 m above sea level; San Pedro (PSP), 18°09′N 96°30′W altitude 76 m above sea level. The Mazateco Honey and Nectar Beekeepers Association provided the samples in San Jerónimo, Oaxaca. Propolis samples were stored in the dark at 15 °C during transportation. Once the propolis samples were received at the food analysis laboratory of the Institute of Agricultural and Livestock Sciences, the samples were finely ground in a blender (Waring Laboratory Science, Model HGB55E, Stamford, CT, USA). They were subsequently frozen in black bags at −4 °C until processing.

### 2.2. Ultrasound-Assisted Extraction

The method described by Oroian et al. [32] was used with some modifications. Ten grams of ground propolis samples were extracted by adding 300 mL of 80% ethanol (1:30); then, the samples were placed in an ultrasonic bath (Branson, 3510R-DTH, Danbury, CT, USA), for 30 min at 25 °C (40 kHz). The sonicated samples were then centrifuged (Hermle, Z 36 HK, Gosheim, Germany) at 6000 rpm for 15 min at 4 °C; subsequently, the supernatant was recovered and filtered, and then they were placed in refractory containers and left in a forced air oven at 40 °C for 24 h for solvent removal. The calculation was carried out using the Equation (1) proposed by [33]:

(1) $$\%\mathrm{yield}=\frac{\mathrm{Extract}\mathrm{weight}\;(g)}{\mathrm{Raw}\mathrm{propolis}\mathrm{weight}\;(g)}\times 100$$

### 2.3. Determination of Bioactive Compounds

#### Determination of Total Phenolic Content

The polyphenol test was performed based on Singleton et al. [34] but slightly modified. A total of 0.5 mL of propolis extract and 2.5 mL of Folin–Ciocalteau (F9252, Sigma-Aldrich, St. Louis, MO, USA) were mixed for 7 min in the dark. Then, 2 mL of 7.5% sodium carbonate (Na_2_CO_3_) (PQ17881 Fermont, Monterrey, Mexico) was added. The tubes with the mixture were incubated for 2 h at room temperature in the dark, and the absorbance was immediately measured at 760 nm in a spectrophotometer (JENWAY, Model 6715, Dunmow, UK). Distilled water was used as the blank. A standard curve was prepared with a gallic acid stock solution (G7384 Sigma-Aldrich, St. Louis, MO, USA). The results were expressed in mg gallic acid equivalents/100 g (mg GAE/100 g). The calculation was conducted using the Equation (2):

(2) $$\mathrm{Total}\mathrm{phenols}\;(\frac{mgEAG}{100g})=\frac{CAG*V*DF*100}{\mathrm{Weight}\mathrm{of}\mathrm{raw}\mathrm{propolis}\;(g)}$$

where

CAG = gallic acid concentration standard curve (mg/mL)
V = sample volume (mL)
DF = sample dilution factor

The method described by Espinoza-Muñoz et al. [35] was modified to determine total flavonoid content. For this determination, 0.5 mL of propolis extract was taken, and 75 μL of 5% sodium nitrite (NaNO_2_) (Meyer, Mexico City, Mexico) was added and allowed to rest for 5 min at room temperature. Subsequently, 150 µL of 10% aluminum trichloride (AlCl_3_) (449598 Sigma-Aldrich St. Louis, MO, USA) was added and left again for 6 min. Then, 500 µL of 1 M sodium hydroxide (NaOH) (Meyer, Mexico City, Mexico) and 275 µL of distilled water were added. The reading was then carried out at 415 nm in a spectrophotometer (JENWAY, Model 6705 Staffordshire, UK). A quercetin standard curve (1592409 Sigma-Aldrich, St. Louis, MO, USA) was used. The results were expressed in mg quercetin equivalents/100 g (mg QE/100 g). The calculation was carried out using Equation (3):

(3) $$\mathrm{Total}\mathrm{flavonoids}\;(\frac{mgEQ}{100g})=\frac{CQ*V*DF*100}{\mathrm{Weight}\mathrm{of}\mathrm{raw}\mathrm{propolis}\;(g)}$$

where

CQ = quercetin concentration standard curve (mg/mL)
V = sample volume (mL)
DF = sample dilution factor

### 2.4. Determination of Antioxidant Activity

#### 2.4.1. ABTS^•+^ Free Radical Inhibition Activity

Antioxidant activity was determined using the acidic chromogenic compound 2,2’-azino-bis (3-ethylbenzothiazoline-6-sulfonic acid) (ABTS^•+^) according to Pimentel-González et al. [36]. For the extracts, a propolis extract (1:1000 *w*/*v*) was diluted using 85% ethanol; the samples were homogenized until complete solubility. Subsequently, the extracts were centrifuged at 18,510× *g* for 15 min at 4 °C (Hermle, Z 36 HK, Gosheim, Germany). A total of 20 mL of 7 mM ABTS^•+^ stock solution was prepared (36 mg of ABTS^•+^ reagent was added to 10 mL with distilled water, and 10 mL of 2.45 mM potassium persulfate (5.83 mg) was added to the potassium persulfate in 10 mL of distilled water). The mixture was kept under magnetic stirring for 24 h in total darkness to generate ABTS^•+^ free radicals. A total of 0.2 mL of the supernatant with 2 mL of standardized ABTS^•+^ was placed in a test tube, homogenized, and allowed to rest at room temperature for 6 min without light. The samples were read at 734 nm on a spectrophotometer (Jenway 6715, Staffordshire, UK). Gallic acid was used as a blank to prepare the standard curve (0 to 100 mg/L)—20% ethanol. According to the following equation, the antioxidant activity was expressed as mg equivalents of gallic acid in 100 g of extract. All samples were analyzed in triplicate. The calculation was carried out using Equation (4):

(4) $$\mathrm{Antioxidant}\mathrm{activity}\;(\frac{mgAG}{100g})=\frac{AG*V*DF*100}{\mathrm{Gross}\mathrm{weight}\mathrm{of}\mathrm{propolis}\;(g)}$$

where

AG = gallic acid concentration standard curve (mg/mL)
V = sample volume (mL)
DF = sample dilution factor

#### 2.4.2. DPPH^•^ Free Radical Inhibition Activity

The determination of antioxidant activity by DPPH^•^ (1,1-diphenyl-2-picrylhydrazyl radical) was according to Brand-Williams et al. [36]. In the case of the extracts, a propolis extract (1:1000 *w*/*v*) was diluted using pure methanol, and the samples were homogenized until they were completely soluble. Subsequently, the extracts were centrifuged at 18,510× *g* for 15 min at 4 °C (Hermle, Z 36 HK, Gosheim, Germany). A total of 100 mL of 0.2 mM DPPH^•^ stock solution was prepared (7.9 mg of DPPH^•^ making up to 100 mL with 80% methanol). It was kept under magnetic stirring for 2 h in total darkness. Before reading the extracts, the DPPH^•^ reagent was standardized to an absorbance of 0.7 + 0.01 using 80% alkaline methanol. A total of 2.5 mL of the standardized DPPH^•^ reagent was added to 0.5 mL of the supernatant and incubated for 1 h without light. The samples were read at 517 nm with a spectrophotometer (Jenway 6715, Staffordshire, UK), and methanol was used as the blank. According to the following equation, the antioxidant activity was expressed as mg equivalents of gallic acid in 100 g of extract and emulsion. All samples were analyzed in triplicate. The calculation was carried out using Equation (5):

(5) $$\mathrm{Antioxidant}\mathrm{activity}\;(\frac{mgAG}{100g})=\frac{AG*V*DF*100}{\mathrm{Gross}\mathrm{weight}\mathrm{of}\mathrm{propolis}\;(g)}$$

where

CAA or AG = standard curve gallic acid concentration (mg/mL)
V = sample volume (mL)
DF = sample dilution factor

### 2.5. Enzyme Inhibition Assays 

#### 2.5.1. In Vitro ɑ-Amylase Assay

The amount of reducing sugars was quantified using the Miller method, slightly modified from Abirami et al. [37], to measure the percentage of inhibition of the α-amylase enzyme. The objective was to measure the amount of reducing sugars in the sample due to the hydrolysis activity of α-amylase on the substrate. A total of 100 µL of propolis extract was taken and mixed with 100 µL of buffer. A total of 0.02 mol/L of sodium phosphate (pH 6.9) and 100 µL of a solution of α-amylase (*Bacillus licheniformis* α-amylase, 1 U/mL, A3306 Sigma-Aldrich, St. Louis, MO, USA) were pre-incubated at 37 °C for 10 min; subsequently, 100 µL of 0.1% soluble starch was added. The samples were incubated at 37 °C for 60 min; the reaction was stopped by adding 1 mL of DNS reagent (1 g of 3,5-dinitrosalicylic acid, 20 mL of a 2 mol/L NaOH solution, 50 mL of distilled water, and 30 g of Rochelle salt). The tubes with the samples were heated in a water bath at 80 °C for 5 min and then placed in a cold-water bath. A total of 3 mL of distilled water was added, and the absorbance of the samples was measured at 540 nm. The readings were compared to the negative control in which the buffer replaced the enzyme inhibitor. The enzyme inhibition was calculated as a percentage. Acarbose (2 mM) was a positive control [38]. Previously, a dextrose calibration curve was prepared to quantify the amount of reducing sugars obtained due to the hydrolysis of starch by the action of α-amylase.

Equation (6), described by [39], used phosphate buffer as a control, where the sample Abs is the absorbance obtained from the samples and control Abs is the absorbance using the phosphate buffer:

(6) $$\%\mathrm{Percentage}\mathrm{of}\mathrm{inhibition}\mathrm{of}ɑ-\mathrm{amylase}=\frac{Abs_{\mathrm{control}}-Abs_{\mathrm{muestra}}}{\mathrm{Abs}\mathrm{Control}}$$

#### 2.5.2. In Vitro ɑ-Glucosidase Assay

The inhibition potential of the propolis extracts on the enzyme α-glucosidase was evaluated by measuring the formation of 4-nitrophenol as a result of the interaction between the enzyme and the substrate 4-nitrophenyl α-D-glucopyranoside (pNPG), according to [40] with modifications. Ten µL of propolis extract was added to 2.6 mL of 0.05 mol/L sodium phosphate buffer (pH 6.9) and 25 µL of an α-glucosidase solution; then, the mixture was incubated at 37 °C for 10 min. Subsequently, 25 µL of pNPG substrate was added. The samples were set at 37 °C for 40 min. The reaction was stopped by adding 25 µL of sodium carbonate solution (0.1 M). The absorbance was measured at 405 nm. The readings were compared to the positive control. The enzyme inhibition was calculated as a percentage. Acarbose was used as a positive control. All assays were performed in triplicate. The amount of p-nitrophenol released during the assay was quantified to determine the activity of the α-glucosidase enzyme. Changes in the hydrolysis of pNPG to pNP by the action of the enzyme were measured. The α-glucosidase enzyme hydrolyzes the colorless substrate, producing free p-nitrophenol. The calculation was carried out using Equation (7):

(7) $$\%\mathrm{Percentage}\mathrm{of}\mathrm{inhibition}\mathrm{of}ɑ-\mathrm{glucosidase}=\frac{Abs_{\mathrm{control}}-Abs_{\mathrm{muestra}}}{\mathrm{Abs}\mathrm{Control}}$$

### 2.6. In Vitro Digestion

(a) Gastric phase: A total of 10 mL of the extracts and control (distilled water) was used; a total of 15 mL of gastric fluid was added (2000 U/mL of porcine pepsin in 0.3 M CaCl_2_ in 0.1 M HCl), the pH was adjusted to 2 with HCl 6 M, the resulting mixture was incubated in a water bath at 37 °C, and horizontal shaking was maintained for 120 min. (b) Intestinal phase: The pH was adjusted to 7 with 0.5 M sodium bicarbonate; subsequently, 10% of pancreatic fluid was added (0.4 g of pancreatin and 2.5 g of bile salts in 100 mL of NaHCO_3_ 0.1 M); the mixture was incubated in a water bath at 37 °C with shaking for 120 min. Finally, the mixture was heated to boiling for 4 min to inactivate the enzymes. Samples were taken at the end of each phase (gastric and intestinal). All samples were centrifuged (Hermle, Z 36 HK, Gosheim, Germany) at 18,510× *g* for 10 min at 4 °C. Subsequently, they were stored in the refrigerator until analysis: total phenolic content, total flavonoid content, and antioxidant activity (ABTS^•+^ and DPPH^•^).

### 2.7. Analysis of Results

The results were processed with analysis of variance (ANOVA). Differences between sample means were analyzed using a Tukey mean comparison test when significant differences were found (*p* < 0.05). The expression of results was given as mean ± standard deviation of tests performed in triplicate. All analyses were performed using IBM^®^ SPSS Statistics version 24 software.

## 3. Results and Discussions

### 3.1. Extract Yield Percentage

Table 1 shows significant differences (*p* < 0.05) between the percentages of extraction yield. When comparing the phenolic content, the propolis from Eloxochitlán (PE) and San Pedro (PSP) obtained higher yields than those from Teotitlán (PT) and San Jerónimo (PSJ). Ororian et al. [32] obtained similar results when extracting propolis from Romania; they reported 32% to 92% extracting yields. Tran et al. [41] reported 19 to 69% yields for Australian propolis. In another study by [42], eight samples from different states of Mexico were analyzed; the best extraction yield was 50.9%, and the minimum was 25.5%. The variation in yields could be due to poor beekeeping practices. When raw propolis is extracted, it contains impurities (wax, insect remains, and other debris). Furthermore, the extraction yield could also be affected by the extraction method, solvent type, extraction time, and temperature [43,44].

### 3.2. Bioactive Compounds during Digestion

The total phenolic content of the propolis samples presented significant differences (*p* < 0.05) in a range from 15,362.4 to 11,520.3 mg GAE/100 g (Table 2). These results are lower than those presented by [23], where a sample of propolis from China was studied, which reported 24,584 mg GAE/100 g; on the other hand, Zainal et al. [45] reported ranges from 7621 to 9294 mg GAE/100 g for propolis from Malaysia. Bees collect resin from different plants, so the variability of the chemical compounds in propolis could be directly related to the availability of the vegetation where the hive is located [46]. One of the main factors in obtaining bioactive compounds is the extraction method, which is studied and compared, concluding that ultrasound extraction is the best alternative by establishing the appropriate conditions [20,47,48]. The PT extract stood out for its flavonoid content with 8084.6 mg QE/100 g, while the PE, PSP, and PSJ extracts had a content between 3721.7 and 5881.6 mg QE/100g (Table 2). Aboulghazi et al. [49] analyzed Moroccan propolis. They quantified lower values that ranged between 970 and 3472 mg QE/100 g under the same extraction time but different ratios and concentrations of solvent; likewise, [50,51] they carried out studies using extraction with maceration, where they quantified total flavonoid content from 1745 to 2479 mg QE/100 g and 1 to 1298 mg QE/100 g (Colombian propolis), respectively. Propolis extracts have different chemical compositions and biological activity due to a correlation between the flora and the extraction method, and it could be influenced by the environmental difference between regions and the collection time [52,53]. 

Two-phase digestion (gastric and intestinal) was carried out on the propolis extracts. It was observed that the PE extract was the one with the highest total phenolic content, followed by PT, PSJ, and PSP; a similar content was measured after the gastric phase, ranging from 6022.0 to 3658.2 mg GAE/100 g, and then after the intestinal phase, ranging from 3284.8 to 1713.8 mg GAE/100 g. PE had the highest content and lowest decrease in phenols in contrast to PSP, which had the lowest range and highest drop (Table 2. González et al. [54] reported a similar behavior with an 80% reduction in the range of total phenolic content. The above is related to studies where a decrease in pepsin activity has been observed, suggesting autolytic cleavages occur after digestion activation.

**Figure 1:**
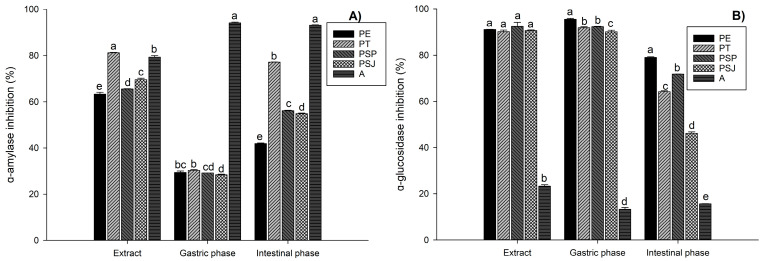
The enzymatic inhibition of propolis extracts in simulated digestion. (**A**) ɑ-amylase inhibition, (**B**) ɑ-glucosidase inhibition. Eloxochitlán propolis (PE), Teotitlán propolis (PT), San Pedro propolis (PSP), San Jerónimo propolis (PSJ), and acarbose (**A**). Values presented are the mean ± standard error of tests performed in triplicate. Letters indicate significant differences (*p* < 0.05) as determined with Tukey comparison of means.

On the other hand, phenolic compounds can covalently bind to protein structures, such as enzymes and cellular receptors [14,55]. Therefore, it supposes an interaction between the chemical compounds and pepsin or its fragments in addition to acidic pH since it induces acid hydrolysis or structural transformation of phenolic compounds [56]. According to the flavonoid content, the extracts can be ordered from highest to lowest, PT, PSJ, PE, and PSP (Figure 1), during the gastric and intestinal phases; this could be because the same conditions were used, and there are significant differences (*p* < 0.05) between them. The total flavonoid content showed a decrease at the end of the gastric phase (80–96%), but after the intestinal phase, it slightly increased (66–79%) (Table 2). Ozdal et al. [57] analyzed 11 propolis extracts with simulated digestion and observed a similar behavior as the digestion progressed, obtaining a decrease in the flavonoid content of the extract in the intestinal phase of up to 45%, while González et al. [54] reported a reduction of 92%. Chen et al. [43] conducted an in vitro digestion model following the established method for assessing carotenoid bioavailability. In the evaluation of nine commercially available tea juices, a notable reduction in total polyphenol content was observed in five of the juices during the gastric phase. However, after the duodenal phase, four of the juices exhibited a subsequent increase in total polyphenol content, suggesting a potential structural transformation of polyphenols during this phase.

#### 3.2.1. Determination of Antioxidant Activity during Digestion

Table 3 shows that the PE extract has greater antioxidant capacity through the inhibition of the ABTS^•+^ radical with 33,307.1 mg ET/100 g; the content in the PSJ > PSP > PE extracts are in a range from 13,984.4 to 25,483.4 mg ET/100 g, and there are significant differences between the extracts (*p* < 0.05). The PT extract has more significant inhibition of the DPPH^•^ radical than the PE extract, while the PSP and PSJ samples have no significant difference (*p* < 0.05). The results obtained from ABTS^•+^ are similar to those reported by [58,59], where they reported 18,640 mg ET/100 g and 21,135 mg ET/100 g, respectively. Peng et al. [60] reported maximum values of DPPH^•^ and ABTS^•+^ of 11,845 mg of TE/100 g and 17,518 mg of TE/100 g, respectively. They were higher than those reported in this study; likewise, Altuntaş et al. [61] reported DPPH^•^ values that ranged between 4.672 and 22.823 mg TE/100 g, where they used propolis from Turkey. The variation in the high antioxidant potential in inhibiting the ABTS^•+^ and DPPH^•^ radicals is mainly due to the presence of different phenolic compounds and flavonoids in the propolis extracts [62,63,64].

In the digestion, there was a decrease in the elimination potential of the ABTS^•+^ radical of the extract to the gastric phase from 64 to 93%, and the DPPH^•^ radical was from 58 to 78%; likewise, the extracts showed a decrease in the inhibition of the radical ABTS^•+^ to the intestinal phase of PE 87%, PT 88%, PSP 94%, and PSJ 76%, and in the elimination of the DPPH^•^ radical, the decrease was PE 55%, PT 74%, PSP 53%, and PSJ 45%. Similar results were reported by [57], wherein the DPPH^•^ had an increase from the gastric phase to the intestinal phase; however, González et al. [54] reported decreases in the antioxidant activity of the ABTS^•+^ radical in the gastric phase from 86 to 93% and, in the DPPH^•^ radical, reported an increase of almost 50%, which they attributed to the acidic pH; in this study, this increase was not observed because it was neutralized to avoid pH interference, and thus, phenolic compounds do not act as pro-oxidants [65]. On the other hand, the antioxidant activity depends on the chemical composition of propolis, which has a great diversity of these compounds and can change the behavior of the simulated digestion due to the transformations of the phenolic compounds that depend on the pH, such as degradation, epimerization, hydrolysis, and oxidation [66].

#### 3.2.2. Determination of Antidiabetic Activity during Digestion

The inhibition of ɑ-amylase showed that acarbose has an inhibition of 79.3%. However, in the extracts, it was PT (81.2%) and PSJ (69.5%), while the others remained above 60% (Figure 1A). It has been reported in other studies that flavonoids are the main antidiabetic agents in propolis [56]; this relationship is noted because PT and PSJ are also the extracts with the highest flavonoid content. In the ɑ-glucosidase assay, acarbose has the lowest inhibition with 23%, followed by propolis extracts, which do not present a significant difference and have an average inhibition of 91% (Figure 1B). Karagecili et al. [67] reported that acarbose has less inhibition of ɑ-glucosidase than Turkish propolis. These results are similar to previous reports, which showed inhibition of ɑ-amylase and ɑ-glucosidase in Nigerian propolis [68], propolis from Morocco [69], Turkish propolis [70], and Australian propolis [71], and which concluded that phenolic compounds and mainly flavonoids can inhibit these digestive enzymes, which delays the digestion of carbohydrates and prevents the absorption of glucose, therefore reducing postprandial hyperglycemia.

All extracts showed antidiabetic activity in the two phases of digestion; in the ɑ-amylase assay, it was observed that there was a decrease in the gastric phase in the four propolis, followed by an increase in the intestinal phase; on the other hand, when moving from the extract to the intestinal phase, propolis had a decrease from 14 to 33% (Figure 1A). In the ɑ-glucosidase assay, it was observed that its inhibition was maintained in the gastric phase. Finally, when moving from the extract to the intestinal phase, they had a decrease in PE (13%), PSJ (22%), PT (28%), and PSP (49%). Because the inhibition of enzymes is attributed to phenolic compounds and propolis has a large amount and variety of these compounds, its behavior in simulated digestion changes in each sample and resembles the behavior of phenols during digestion [72]. The fact that there are no changes in inhibition of the extract in the gastric phase may be because it has a higher content of phenols and flavonoids; these, when passing through the acidic medium, maintain their inhibition; similar results were reported by [73], where they evaluated the effect of in vitro digestibility on the enzymatic activity in honey, where their activity decreased in each phase of digestion. On the other hand, Medina-Pérez et al. [74,75] analyzed acidic cactus extract and its encapsulation and reported that the inhibition decreased or was maintained when passing through the digestion phases.

Pearson correlation coefficients of the gastric phase data (Supplementary Figure S1) showed that ɑ-amylase inhibition percentage and total flavonoid content were correlated (r = 0.58, *p* < 0.05). The ɑ-glucosidase inhibition percentage also correlated with total phenol content (r = 0.68, *p* < 0.05). Regarding the Pearson correlation coefficients of the intestinal phase data (Supplementary Figure S2), it was observed that ɑ-amylase inhibition percentage and total flavonoid had r = 0.46, (*p* < 0.05). Finally, ɑ-glucosidase observed a negative correlation with total flavonoid content (r = −0.71, *p* < 0.01). Phenolic compounds can hinder the functions of carbohydrate-hydrolyzing enzymes by interacting with proteins [76]. It is evident that distinct aqueous ethanol extracts of propolis exhibit varied inhibition patterns against ɑ-amylase and α-glucosidase enzymes [77]. El-Guendouz et al. [69] found a noteworthy connection between the overall phenolic content, total flavonoid content, and the activity of α-amylase in a study involving twenty-one Moroccan propolis samples. The above suggests that phenolic compounds play a substantial role in influencing α-amylase activity [78].

## 4. Conclusions

The extraction yields of bioactive compounds from Mexican propolis extracts obtained with ultrasound are not related to their phenolic compound content; the origin of the samples influences them as well as the proportions of resin and other compounds. The polyphenol content is associated with its antioxidant and antidiabetic activity. The present work studied the effects of simulated digestion on aqueous ethanolic extracts of Mexican propolis. A reduction in the total content of phenols and flavonoids was observed as an effect of the digestive phases on the extracts. However, the percentage of inhibition of the digestive enzymes (ɑ-amylase and ɑ-glucosidase) is maintained and increased. Our findings seem to demonstrate that the glycemic control of propolis may be related to the inhibitory activities against glucosidase. Therefore, aqueous propolis extracts in ethanol can be used as nutraceuticals to regulate postprandial hyperglycemia. In the future, it will be necessary to protect the bioactive compounds of propolis during the processing and digestion stages in the food industry for their application in various food matrices.

## Figures and Tables

**Table 1:** Percentage yield of propolis.

Propolis Samples	% Extraction Yield
Eloxochitlan (PE)	68 ± 0.28 ^b^
Teotitlan (PT)	42 ± 0.25 ^d^
San Pedro (PSP)	69 ± 0.35 ^a^
San Jeronimo (PSJ)	49 ± 0.26 ^c^

Values presented are the mean ± standard error of tests performed in triplicate. Letters indicate significant differences (*p* < 0.05) as determined with Tukey comparison of means.

**Table 2:** Total phenolic and total flavonoid content in propolis extracts during the gastric and intestinal phases in an in vitro digestion.

	TPC (mg GAE/100 g)			TFC (mg QE/100 g)		
	Extract	Gastric Phase	Intestinal Phase	Extract	Gastric Phase	Intestinal Phase
PE	15,362.4 ± 225 ^a^	6022.0 ± 171 ^a^	3284.8 ± 148 ^a^	4395.5 ± 62 ^c^	383.6 ± 39 ^c^	1524.3 ± 60 ^c^
PT	12,360.8 ± 158 ^b^	4464.9 ± 265 ^bc^	2594.5 ± 357 ^b^	8084.6 ± 19 ^a^	1481.1 ± 55 ^a^	2146.5 ± 37 ^a^
PSP	11,520.3 ± 247 ^c^	3658.2 ± 485 ^c^	1713.8 ± 4 ^c^	3721.7 ± 73 ^d^	131.2 ± 23 ^d^	807.0 ± 51 ^d^
PSJ	11,820.5 ± 455 ^bc^	4521.2 ± 265 ^b^	2439.0 ± 214 ^bc^	5881.6 ± 27 ^b^	588.4 ± 53 ^b^	1958.0 ± 33 ^b^

Total phenolic content (TPC), total flavonoid content (TFC), Eloxochitlán propolis (PE), Teotitlán propolis (PT), San Pedro propolis (PSP), and San Jerónimo propolis (PSJ). Values presented are the mean ± standard error of tests performed in triplicate. Letters indicate significant differences (*p* < 0.05) as determined with Tukey comparison of means.

**Table 3:** Antioxidant capacity in propolis extracts during the gastric and intestinal phases in in vitro digestion.

	ABTS^•+^ (mg ET/100 g)			DPPH^•^ (mg ET/100 g)		
	Extract	Gastric Phase	Intestinal Phase	Extract	Gastric Phase	Intestinal Phase
PE	33,307.1 ± 567 ^a^	4867.0 ± 136 ^b^	4068.7 ± 360 ^a^	3242.3 ± 69 ^b^	839.2 ± 39 ^ab^	1454.7 ± 22 ^a^
PT	13,984.4 ± 436 ^d^	1546.5 ± 104 ^c^	1587.1 ± 86 ^b^	3611.4 ± 17 ^a^	764.2 ± 8 ^b^	907.7 ± 33 ^d^
PSP	25,483.4 ± 601 ^b^	1592.0 ± 68 ^c^	1471 ± 132 ^b^	2250.1 ± 43 ^c^	928.6 ± 54 ^a^	1052.2 ± 66 ^c^
PSJ	17,041.1 ± 327 ^c^	6004.6 ± 208 ^a^	3924.5 ± 259 ^a^	2296.3 ± 59 ^c^	940.2 ± 39 ^a^	1246.1 ± 28 ^b^

Eloxochitlán propolis (PE), Teotitlán propolis (PT), San Pedro propolis (PSP), and San Jerónimo propolis (PSJ). Values presented are the mean ± standard error of tests performed in triplicate. Letters indicate significant differences (*p* < 0.05) as determined with Tukey comparison of means.

## Data Availability

The original contributions presented in the study are included in the article, further inquiries can be directed to the corresponding author.

## Supplementary Materials

The following supporting information can be downloaded at: https://www.mdpi.com/article/10.3390/foods13020348/s1, Figure S1. Pearson correlation coefficients graph of data from analysis carried out on samples from the gastric phase; Figure S2. Pearson correlation coefficients graph of data from analysis carried out on samples from the intestinal phase; Figure S3. Pearson correlation coefficients graph of the analyzes carried out on the propolis extracts.
