# Supplementary material for: Potential Antidiabetic Activity of Apis mellifera Propolis Extraction Obtained with Ultrasound
Source: Foods. 2024 Jan 22;13(2):348. doi: 10.3390/foods13020348 (PMC10815508; doi:10.3390/foods13020348)
Supplement: Supplementary file 1 [file foods-13-00348-s001.zip › foods-2740496-supplementary.pdf]

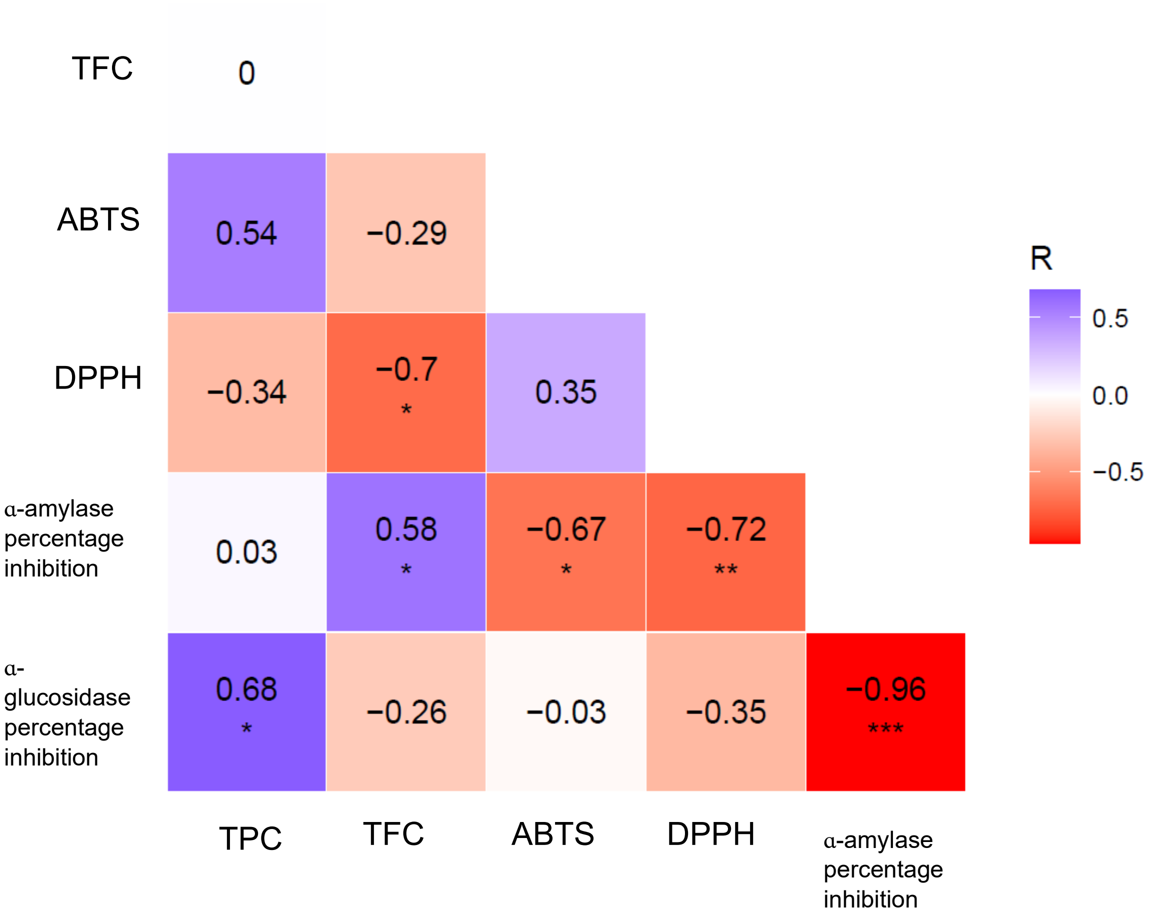

*p<0.05; **p<0.01; and ***p<0.001

Figure S1. Pearson correlation coefficients graph of data from analysis carried out on samples from the gastric phase.

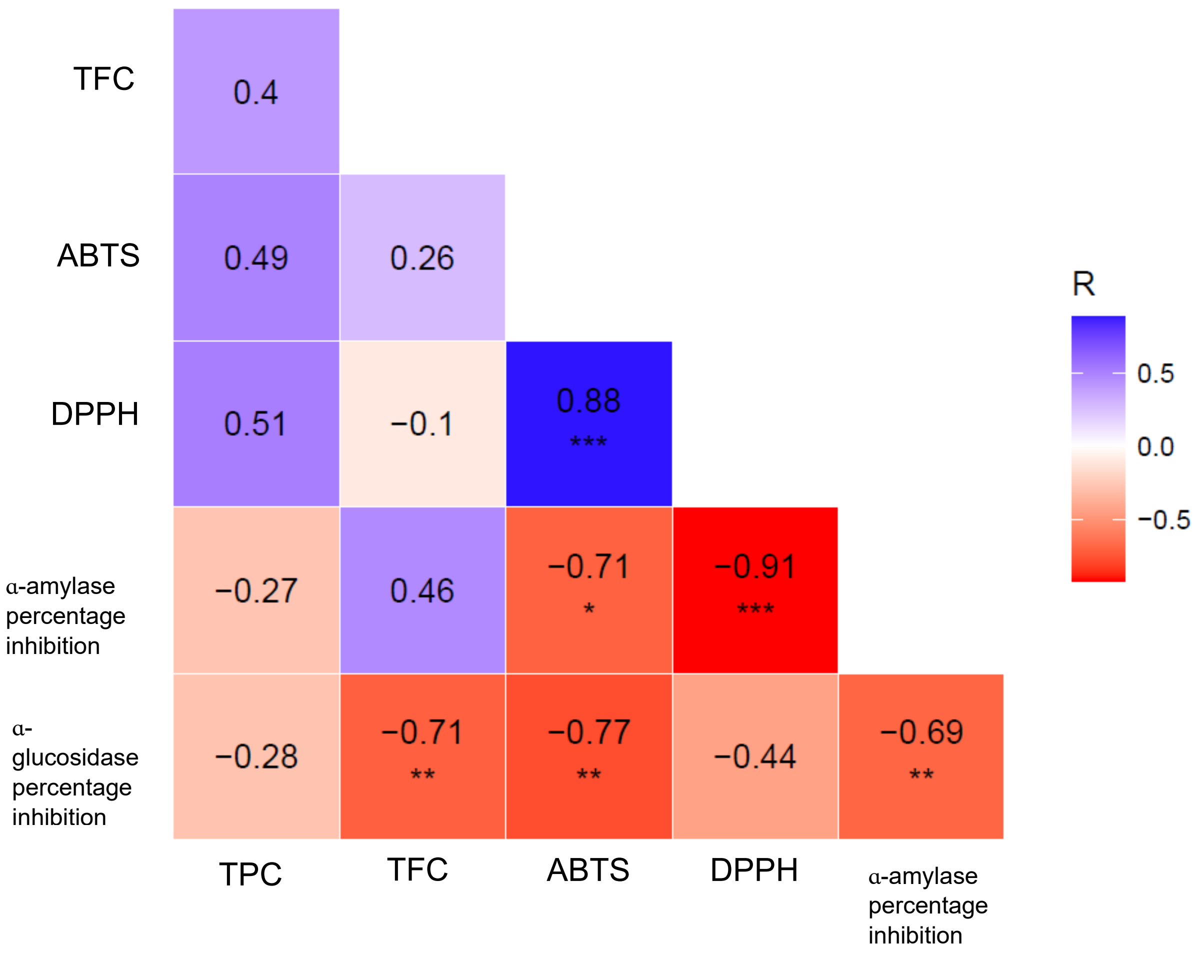

p<0.05; **p<0.01; and ***p<0.001

Figure S2. Pearson correlation coefficients graph of data from analysis carried out on samples from the intestinal phase.

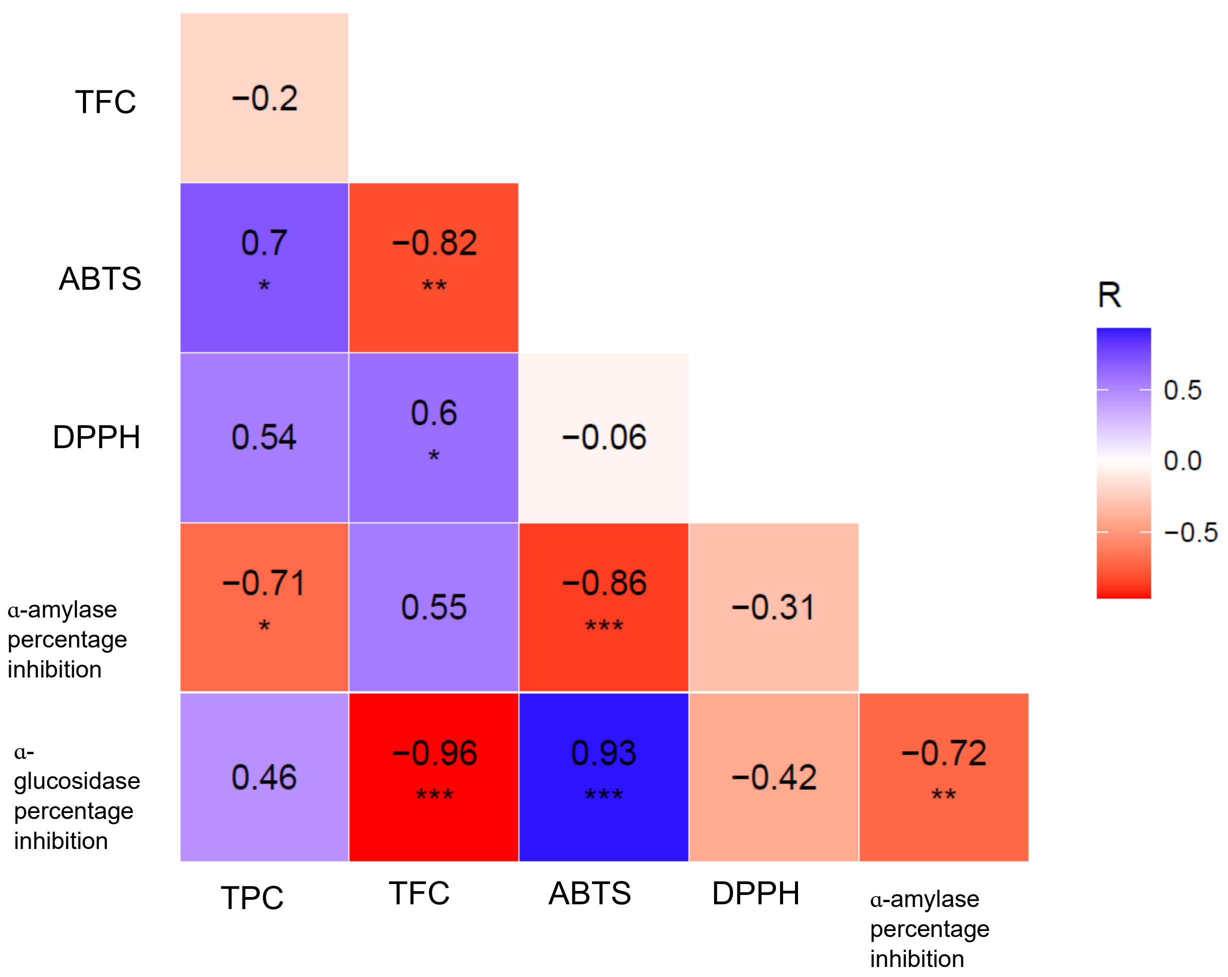

*p<0.05; **p<0.01; and ***p<0.001

Figure S3. Pearson correlation coefficients graph of the analyzes carried out on the propolis extracts.
